# Imbalanced basal ganglia connectivity is associated with motor deficits and apathy in Huntington’s disease

**DOI:** 10.1093/brain/awab367

**Published:** 2021-10-11

**Authors:** Akshay Nair, Adeel Razi, Sarah Gregory, Robb B Rutledge, Geraint Rees, Sarah J Tabrizi

**Affiliations:** 1 Huntington’s Disease Centre, UCL Queen Square Institute of Neurology, University College London, Russell Square House, London WC1B 5EH, UK; 2 Max Planck UCL Centre for Computational Psychiatry and Ageing Research, UCL Queen Square Institute of Neurology, University College London, Russell Square House, London WC1B 5EH, UK; 3 Turner Institute for Brain and Mental Health, Monash Biomedical Imaging, Monash University, Clayton 3800, Australia; 4 Wellcome Centre for Human Neuroimaging, UCL Queen Square Institute of Neurology, University College London, London WC1N 3AR, UK; 5 Department of Psychology, Yale University, New Haven, CT 06511, USA; 6 UCL Institute of Cognitive Neuroscience, University College London, Alexandra House, Bloomsbury, London WC1N 3AZ, UK

## Abstract

The gating of movement depends on activity within the cortico-striato-thalamic loops. Within these loops, emerging from the cells of the striatum, run two opponent pathways—the direct and indirect basal ganglia pathways. Both are complex and polysynaptic, but the overall effect of activity within these pathways is thought to encourage and inhibit movement, respectively. In Huntington’s disease, the preferential early loss of striatal neurons forming the indirect pathway is thought to lead to disinhibition, giving rise to the characteristic motor features of the condition. But early Huntington’s disease is also associated with apathy, a loss of motivation and failure to engage in goal-directed movement. We hypothesized that in Huntington’s disease, motor signs and apathy may be selectively correlated with indirect and direct pathway dysfunction, respectively.

We used spectral dynamic casual modelling of resting-state functional MRI data to model effective connectivity in a model of these cortico-striatal pathways. We tested both of these hypotheses *in vivo* for the first time in a large cohort of patients with prodromal Huntington’s disease. Using an advanced approach at the group level we combined parametric empirical Bayes and Bayesian model reduction procedures to generate a large number of competing models and compare them using Bayesian model comparison. With this automated Bayesian approach, associations between clinical measures and connectivity parameters emerge *de novo* from the data.

We found very strong evidence (posterior probability > 0.99) to support both of our hypotheses. First, more severe motor signs in Huntington’s disease were associated with altered connectivity in the indirect pathway components of our model and, by comparison, loss of goal-direct behaviour or apathy, was associated with changes in the direct pathway component.

The empirical evidence we provide here demonstrates that imbalanced basal ganglia connectivity may play an important role in the pathogenesis of some of commonest and disabling features of Huntington’s disease and may have important implications for therapeutics.

## Teaser

Using spectral dynamic causal modelling of resting state fMRI data in patients with prodromal Huntington’s disease, Nair *et al*. show that altered connectivity in the direct and indirect basal ganglia pathways is associated with apathy and motor signs, respectively.

## Introduction

Huntington’s disease is an autosomal-dominant neurodegenerative condition caused by a triplet repeat expansion in the huntingtin (*HTT*) gene on chromosome 4.^1,^^2^ While the aetiology of Huntington’s disease is clear, the pathogenesis of many of the core clinical motor, cognitive and behavioural features of Huntington’s disease remain to be established. Although Huntington’s disease ultimately affects almost the entire brain, early degeneration of the striatum is canonical of this disorder both pathologically and on structural imaging.^3,^^4^ The striatum, however, is not a homogeneous structure. As the input node to the basal ganglia it has a complex anatomy. The medium spiny neurons (MSNs) of the striatum form a wide range of compartments and pathways.^5-7^ For Huntington’s disease, this anatomical complexity is of relevance because the disorder does not affect all striatal MSN populations equally.^8,^^9^ Cortico-striatal connections, which are topographically arranged, form the input to the striatum.^10,^^11^ These cortical projections synapse with MSN populations that fall broadly into two key groups—those forming the direct and the indirect pathways.^12,^^13^ They form unique and complex polysynaptic connections with other basal ganglia structures, such as the globus pallidus, subthalamic nucleus (STN) and substantia nigra.^7^ Overall, these two pathways form opponent channels that regulate thalamic control over cortical activation.^10^ In the motor system the activity of the direct pathway encourages movement, whereas the indirect pathway activity inhibits or reduces movement.^14–16^ Although all MSNs are susceptible to degeneration in Huntington’s disease, those of the indirect pathway appear more susceptible earlier in the disease.^9,^^17,^^18^ Based on these observations it has been hypothesized that changes in connectivity within the indirect pathway would be associated with the emergence of motor signs in Huntington’s disease, which are characterized by erratic, noisy and disinhibited movements such as chorea, dystonia, incoordination and jerky eye movements.^19^ Despite the widespread reference to this hypothesis, we know of no direct neuroimaging evidence supporting it.

Establishing the role of altered basal ganglia connectivity in the pathogenesis of Huntington’s disease may also have a wider clinical relevance beyond simply understanding motor signs. Alongside the motor features of the condition, Huntington’s disease is associated with a marked psychiatric phenotype. Although associated with a range of psychiatric disturbances, there appears to be a unique relationship between Huntington’s disease and the development of apathy. Apathy, the loss of motivation and goal-directed behaviour, is a complex construct with a range of anatomical regions and neurochemical pathways hypothesised to play a role. Apathy is, however, highly prevalent in Huntington’s disease.^20^ Apathy in Huntington’s disease also tracks closely with disease progression even in premanifest and prodromal cohorts.^21^ Despite the high prevalence of apathy in Huntington’s disease, its pathogenesis is poorly understood and treatments are sorely lacking.^22,^^23^

Based on these epidemiological observations closely tying apathy to disease progression in Huntington’s disease, we hypothesized that motor signs and apathy in early Huntington’s disease may also be a feature of basal ganglia pathway dysregulation. However, unlike motor signs, we hypothesized that apathy may instead arise from involvement of the direct basal ganglia pathway. We base this hypothesis on two strands of evidence. First, as alluded to above, activation of the direct pathway MSNs is thought to encourage free operant movement.^14–16^ The lack of free-operant action initiation is a characteristic feature of behavioural apathy and disruption to the direct pathway may hamper this final stage of goal-directed behaviour—the expression of action.^24–26^ Second, computational models of basal ganglia function propose that as a result of the physiological asymmetry in dopamine receptor expression, these pathways not only play opponent roles in motor expression but also in goal-directed behaviour.^27–30^ Dysfunction in this pathway may therefore disrupt both the neural circuits necessary to take goal-direct action and impair the computational value associated with taking an action.

In summary, we sought to test two hypotheses—first, that motor signs in Huntington’s disease would be associated with altered indirect pathway connectivity and second, that apathy in early Huntington’s disease may be associated with change in direct pathway connectivity.

To test these hypotheses, we used a neuroimaging technique to model direct and indirect pathway dysfunction. Canonically, these pathways are distinguished by change in activity that they *cause* within the thalamic nuclei. Within a neuroimaging framework, this causal connectivity is described as effective connectivity.^31^ Here we leverage the difference in both anatomical and effective connectivity to test our key hypotheses. To study effective connectivity, we use a Bayesian framework known as dynamic causal modelling (DCM) to build a simplified model of our pathways of interest.^32–35^ We based the model of these pathways on previous work in Parkinson’s disease but using several technological advances to test our hypotheses.^36^ First, we used spectral DCM, a technique shown to outperform stochastic DCM for resting-state functional MRI data analysis.^37,^^38^ Second, at a group level, we used parametric empirical Bayes (PEB) to model how individual (within-subject) connections relate to between-subject factors such as motor scores.^39^ In this manner, our approach accounts for both expected values and model uncertainty throughout our analysis. Finally, we did not specifically test our hypotheses, but rather allowed an automated Bayesian procedure, called Bayesian model reduction (BMR). This approach allowed us to determine whether our hypothesized correlations between clinical scores and connectivity parameters emerged from the data *de novo.*^39–41^

Here we demonstrate, in a large cohort of patients with prodromal Huntington’s disease from the TRACK-ON HD study, that motor signs and apathy in Huntington’s disease are associated with unique basal ganglia connectivity profiles.^42^ Furthermore, we found that as hypothesized, higher motor scores were associated with connectivity changes in the indirect pathway components of our model. By comparison, higher apathy scores were associated with altered direct pathway connectivity changes.

## Material and methods

### Sample

Data collected as part of the TRACK-ON HD were used in this analysis as previously described.^42^ For this analysis, data from the third (and last) TRACK-ON visit were used. Participants aged below 18 or over 65 were not recruited and participants with major psychiatric, neurological, medical disorder or history of head injury were excluded. Participants with the Huntington’s disease mutation all had ≥40 CAG repeats and a disease burden score of >250 at baseline. The study was approved by the local ethics committees and all participants gave written informed consent according to the Declaration of Helsinki. Sample characteristics are described in Table 1. Of 102 scans that passed quality control, two participants were excluded for antipsychotic use. A further six participants who were left-handed were excluded, leaving data from 94 Huntington’s disease gene carriers in the peri-manifest phase of the disease in this study. Although group differences were not the focus of this study, data from 85 right-handed control participants were also used to replicate baseline network connectivity as described below.

**Table 1:** Sample demographics of 94 Huntington’s disease gene carriers who underwent resting-state functional MRI as part of the TRACK-ON study

	Gene carriers (*n* = 94)
Age, mean (±SD)	45.5 (±8.9)
% Female	50
Mean CAG repeat length	43.1 (±2.3)
TMS	10.5 (±8.5)
Apathy score	10.9 (±6.0)
Depressive scores	6.6 (±6.8)
Number by scanner type (Siemens/Philips)	52/42

Apathy measured using the Baltimore Apathy Scale. Depressive symptoms measured using the Beck Depression Inventory. Spread of Unified Huntington’s Disease Rating Scale Total Motor Score and apathy scores in the Supplementary material. Table shows mean ± SD unless otherwise stated.

### Clinical outcomes

Two primary outcomes were used in this study—Unified Huntington’s Disease Rating Total Motor Score (TMS) and the self-rated Baltimore apathy scale (BAS).^43,^^44^ The motor score assesses the severity of 31 common neurological features such as chorea, dystonia, bradykinesia and oculomotor signs. The maximum score possible is 124. Due to the early stage of disease in these patients, and the relatively mild motor signs in the cohort (mean score 10.5; Table 1), the TMS was used as opposed to specific subscales which would be underpowered. The BAS consists of 14 items with scores ranging from 0 to 42, where a higher score represents a higher degree of apathy. Self-rated apathy scores were used for this analysis. Self- and carer-rated apathy have good interrater reliability, especially in the absence of significant cognitive impairment.^44–46^ To control for the effects of depression, Beck Depression Inventory scores were used as a covariate in the apathy analysis.^47^

The BAS was developed based on expert opinion and comprises 14 items which Chatterjee *et al*.^44^ described as capturing different dimensions of apathy. Unlike the other apathy scales, no clear subtypes of apathy (behavioural, emotional, cognitive, etc.) are identified within the scale; however, the scale is weighted towards behavioural apathy. Each item is scored from 0 to 3 with scores ranging from 0 to 42, with higher scores indicating higher apathy. The interrater agreement between patient- and carer-rated apathy were highest when cognitive impairment was minimal, as would be the case in a study like TRACK-ON HD. Chaterjee *et al*.^44^ described using a median split to categorize patients as being apathetic, which equated to a patient rated score of greater than 15. Based on Supplementary Fig. 2 and Table 1, our sample was therefore not markedly apathetic, although we did see a range of scores even at this early stage in the disease.

### MRI data acquisition

3 T MRI data were acquired at four sites: London, Paris, Leiden and Vancouver. T_1_-weighted image volumes were acquired using a 3D MPRAGE (magnetization prepared rapid gradient echo) acquisition sequence as described by Kloppel *et al*.^42^ For resting-state functional MRI, whole-brain volumes were acquired at a repetition time of 3 s using a T_2_*-weighted echo planar imaging (EPI) sequence with the following parameters: echo time 30 ms, field of view 212 mm, flip angle 80°, 48 slices in ascending order (slice thickness: 2.8 mm, gap: 1.5 mm, in-plane resolution 3.3 × 3 mm) and bandwidth of 1906 Hz/Px. In total 165 volumes were acquired over 8:20 min followed by field map acquisition.

### MRI preprocessing

MRI image preprocessing and quality control were as described in Kloppel *et al*.^42^ In brief, the first four EPI images were discarded to allow for steady-state equilibrium. Images were realigned and underwent inhomogeneity correction where field maps were available. EPI images were co-registered to anatomical images and normalized to MNI (Montreal Neurological Institute) space. Data were smoothed with a 6 mm full-width at half-maximum Gaussian kernel. Data underwent significant quality control as described by Kloppel *et al*.^42^ Manual quality control (QC) along with the use of ArtRepair and tsdiffana were used to assess for significant movement before preprocessing. More details from Kloppel *et al*.^42^ regarding the quality control procedures are described in the Supplementary material.

Additionally, we computed the mean, and cumulative, framewise displacement (FWD) as per Power *et al*.^48^ Although there is no established cut-off, Power *et al*. suggest an FWD of < 0.5 mm. In this study, overall movement was very limited in both groups. In the control group, mean FWD was 0.22 ± 0.15 mm (max in group = 0.88 mm). In the Huntington’s disease gene carrier group, mean FWD was 0.23 ± 0.12 mm (max in group = 0.63 mm). Importantly, there was no significant difference between groups [*t*(177) = 0.10, *P* = 0.9], suggesting that additional movement in the Huntington’s disease group was not excessive. Unsurprisingly, there was a weak correlation between Huntington’s disease motor score and FWD (*r* = 0.36, *P* < 0.01) and no correlation with apathy (*r* = 0.12, *P* = 0.23). During preprocessing this movement is corrected for and as described below the in-scanner movement is controlled for in both the placement of regions of interest and during the extraction of the eigenvariate. There is thus no clear reason to believe that movement would affect one subset of connections above others. Mean FWD and cumulative FWD plots by group and clinical variable are shown in the Supplementary Figs 6 and 7.

### Region of interest specification

**Figure 1:**
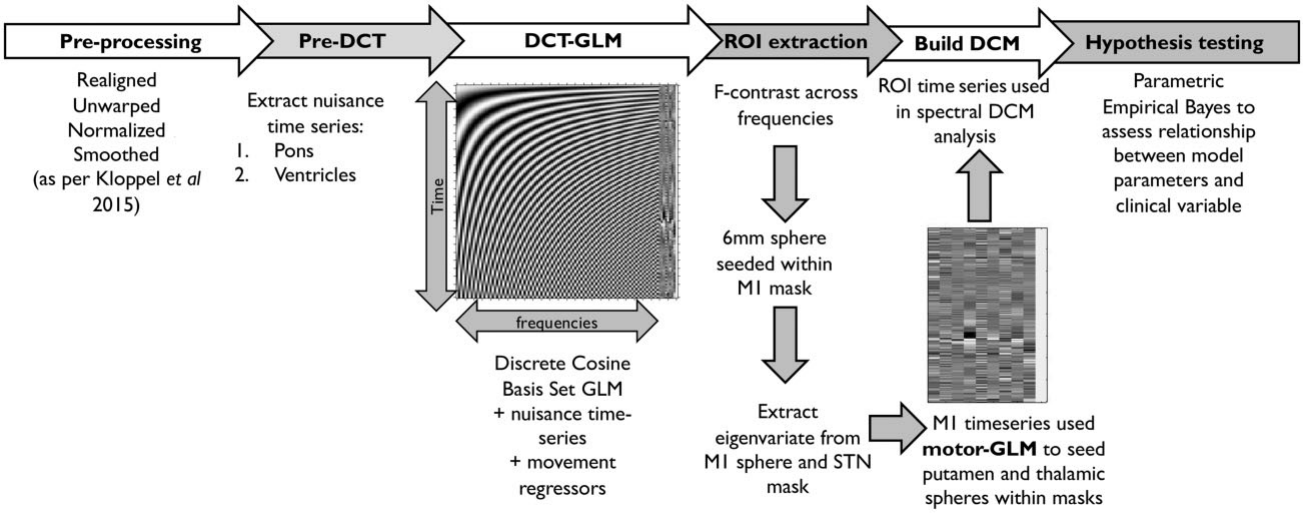
**Summary of the resting state functional MRI analysis pipeline used in this study.** See text for more details.

A summary schematic of the pipeline used to analyse the resting-state functional MRI data is shown in Fig. 1. For this analysis, time series were extracted from four predefined regions of interest to make up the motor basal ganglia loop—the motor cortex, motor thalamus, motor putamen and the STN. With the exception of the STN, time series were extracted from spheres seeded within anatomical masks defined in a standard space. An anatomical mask of Brodmann area 4 from Wake Forest University Atlas was used to define the motor cortex.^49^ The motor putamen and motor thalamus masks were defined from probabilistic connectivity atlases with a threshold of 50% probability.^50,^^51^ The STN was not manually defined in this study. Instead, a mask made available by Keuken *et al*.^52^ was used. This mask, defined in MNI space, was derived from the accurate high-resolution delineation of STN using 7 T imaging, also assessing the impact of age. Based on the sample characteristics in this study (mean age 45.5 ± 8.9), the mask for middle-aged individuals was used again with a conservative threshold of >50% probability. Given the size of this structure and the spatial resolution of functional imaging, the time series extracted may also contain signals from adjacent structures.

### Resting-state functional MRI modelling with GLM

Using the preprocessed scans, a dummy GLM (General Linear Model) was created to extract nuisance time series from the pons and ventricles. To better model resting state low frequency fluctuations, we then used a discrete cosine transform (DCT). In summary, this approach consists of 189 cosine basis functions modelling frequencies in the typical resting state range of 0.0078–0.1 Hz.^36,^^53-56^ We created a GLM containing these DCT regressors as well as the nuisance time series extracted as described above alongside six movement regressors.

An F-contrast was used over the DCT frequencies to identify regions that showed resting state activity within the motor cortex. Based on this contrast, within the BA4 mask, a 6-mm sphere was placed at the location which showed the highest activity in the frequencies of interest. From this sphere the principal eigenvariate (adjusting for head movements and nuisance time series) was extracted. This procedure summarizes the time series from all of the voxels in the sphere into one representative time series for the region of interest. The variance explained by the eigenvariate in the M1 region of interest had a mean of 67% with a variance of ±11.5%. The principal eigenvariate from the entire STN mask was also extracted as above with variance explained mean of 87% with a variance of ±4.3%. The time series extracted from the motor cortex was then used to determine the location of a 4-mm sphere placed within the motor putamen and motor thalamic masks. The centre of these spheres was placed within each mask at the coordinates that showed the strongest correlation with the M1 time series regressor. The principal eigenvariate was extracted from these spheres controlling for the same confounders showing variance explained with a mean of 76% (variance: ±8.6) and 77.3% (variance: ±8.4) in the putamen and thalamus, respectively. Example time series extracted from these regions of interest is shown in Supplementary Fig. 1.

### Dynamic casual modelling and specification of the connectivity matrix

**Figure 2:**
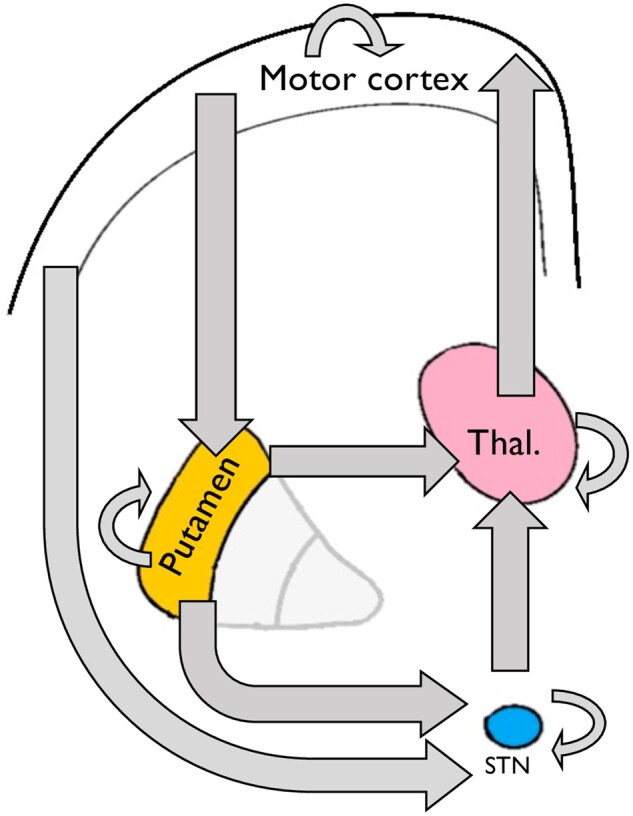
**Schematic of the basal ganglia network modelled in this study (the DCM ‘A-matrix’).** The direction of the arrows indicates that direction of effective connectivity entered into the model. Arrows looping back to the same node represent inhibitory self-connections specified in the DCM.

**Figure 3:**
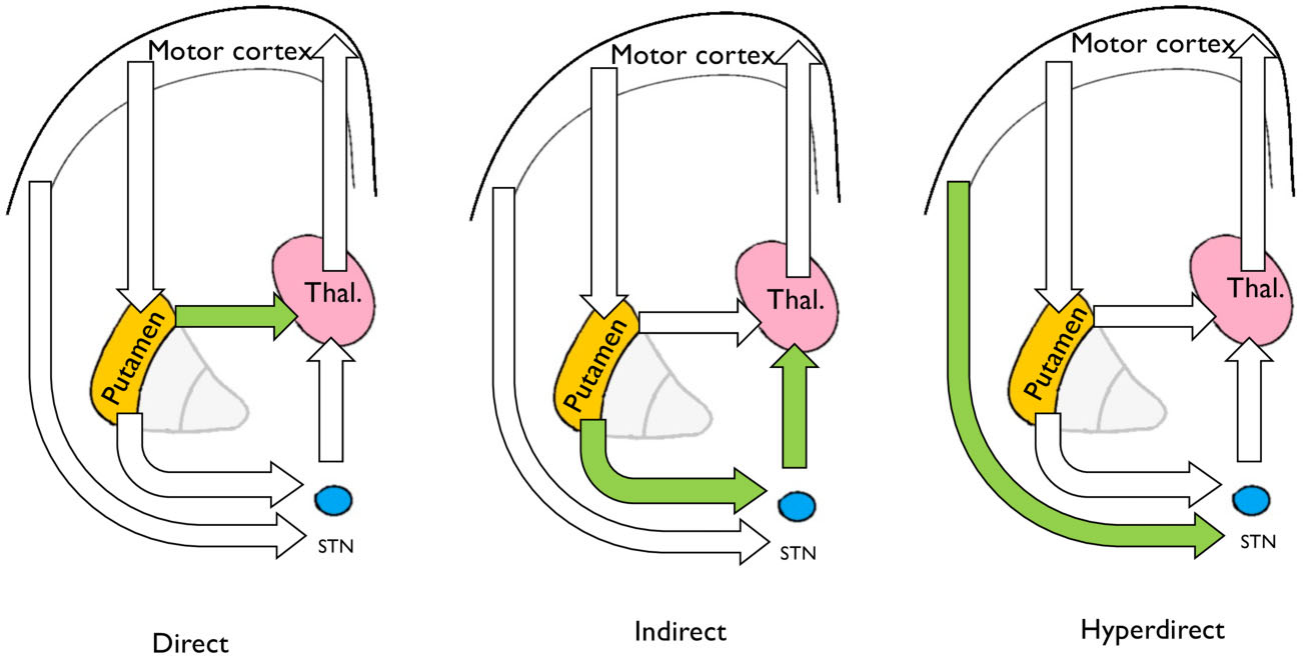
**This model generates simplified representations of three pathways of interest.** (**A**) The direct pathway is composed of the connection between putamen and thalamus. (**B**) The indirect pathway components are the putamen–STN connection and the STN–thalamic connection. (**C**) The hyperdirect pathway comprises a connection from the motor cortex to the STN.

Based on previously published work, we used a simplified circuit representing the direct, indirect and hyper-direct pathway as shown in Fig. 2.^36^ Here we do not model connections involving the globus pallidus; instead, we use a simplified circuit involving motor cortex, putamen, thalamus and STN as described by Kahan *et al*.^36^ A forward connection from M1 to motor putamen represents the input to the network from the motor cortex. The motor putamen was modelled as having two forward connections—one connecting it to the motor thalamus, forming the ‘direct pathway’ of our model, and a second connection linking it to the STN, the first component of the ‘indirect pathway’ of our model. The STN was modelled as having a further connection to the thalamus, forming the second connection within the model’s indirect pathway. A further direct connection between the cortex and the STN was specified representing the hyper-direct pathway. These basal ganglia pathways are shown as a schematic in Fig. 3A–C.

Having specified this network, or *A-matrix*, we used spectral DCM packaged as part of SPM12 to infer effective connectivity parameters. Unlike stochastic DCM, spectral DCM inversion does not predict the time series extracted from each node, but rather estimates their cross-spectral density.^37^ This approach is more accurate at recovering parameters and estimated second-level effects such as group differences.^38^ In DCM, we do not specify the valence of the connections between nodes and allowed these to be estimated from the data. The positive connectivity value refers to an excitatory connection, whereas the negative connectivity value refers to an inhibitory influence.

The true complexity of the basal ganglia circuitry is such that simplification is necessary when using (3 T) functional imaging data. Perhaps the most striking may be that in this work the globus pallidus structures are not modelled, which was also an approach adopted by Kahan *et al*.^36^ This decision was made with two complementary points in mind: (i) to minimize risk of overlap between nodes; and (ii) to limit model complexity while, we argue, retaining the key features of the network.

First, the inclusion of the globus pallidus segments would have necessitated the inclusion of multiple small adjacent nodes from which to extract neural data. While possible, this would have increased the risk of artificial dependencies arising within the data. Second, although perhaps more anatomically accurate, we believe that the inclusion of these nodes would not have added any more clarity to the results. Additional nodes would have resulted in additional components to each pathway. Also, both the direct and indirect pathways drive activity in the globus pallidus; however, it is the (positive) driving effective connectivity between the striatum and thalamus which defines the function of the direct pathway and the inhibition of the thalamus via the STN which defines the indirect pathway. These aspects are modelled using our approach.

### Hypothesis testing with PEB and BMR

The DCM specified above was estimated for each participant separately. The DCMs performed well with variance explained of 83.1% (variance: ±9.7%) in the patient cohort and 83.8% (variance: ±8.4%) in the control cohort. Inference on clinical scores was performed using PEB.^39^ This is a between-participants hierarchical Bayesian model that models how connections at the individual level, such as connectivity parameters, relate to between subject factors, such as motor score. At the first level, individual participant parameters were estimated using spectral DCM. The PEB approach then considered these parameters at the second level as having group means and between-participant variability, which could be explained by between participant factors.

In this procedure, first a parent model—which can be sparse (as here) or fully connected—is estimated in which all regressors of interest such as motor score and covariates are modelled as having an effect on any of the connections specified in the subject-level DCMs. In order to test our hypotheses, we combined this approach with a BMR procedure.^40,^^41^ BMR procedure scores all nested (reduced) models by turning off parameters that do not contribute to the *model evidence*. In brief, BMR enables the (greedy) search of very large model space by scoring each (reduced) model based on model evidence or free energy. The parameters of the best 256 reduced models from this search procedure are then averaged, weighted by their model evidence (i.e. Bayesian Model Averaging). In essence this procedure first assumes that all pathways may be associated with a clinical variable and then proceeds to sequentially remove associations between variables and pathways if they worsen model performance. For further details see refs.^40,^^41^ This procedure derives the posterior densities of the parameters by marginalizing over the models accounting for model uncertainty. In this manner, parameter estimates are not heavily influenced by models with high levels of uncertainty.

Estimates of parameter strength are outputted along with the posterior probability of the parameters being non-zero. These parameters represent the rate of change in activity in the afferent node, measured in Hz, caused by activity in the efferent node. As described by Kahan *et al*.^36^ they can be thought of as the sensitivity of the target node to the source. The PEB models we specified controlled for age, gender and scanner type. The effect of motor score and depressive scores were then additionally controlled for in analyses of apathy in the Huntington’s disease sample. Group comparison was not the focus of this study; however, data from control participants were used to replicate the baseline connectivity profile (as shown in Supplementary material). Regressors were mean-centred, allowing the interpretation of the first covariate of the model to be the average connectivity weights in the network. We only report connections that have a posterior probability of >0.99 (which refers to very strong statistical evidence). Scanner type was modelled as a dummy variable and included as a covariate in every model. There was an effect of scanner on putamen to thalamus connection [−0.4, posterior probability (*pp*) > 0.99]; however, this was controlled for in the results presented.

Having estimated the effect of clinical covariates on connection strengths at a group level, we completed a Bayesian leave-one-out cross-validation procedure, as implemented in SPM, to determine whether these weights could themselves be predictive of an individual participant’s symptom scores. Cross-validation of this sort provides out of sample estimates of predictability (i.e. the predictive validity of the connectivity strength from a new participant).^41^ There is no leakage between the parameters in this analysis as the model is re-estimated with the exclusion of the test case.

### Data availability

Data will be shared on reasonable request post publication.

## Results

### Sample demographics

Our sample consisted of Huntington’s disease gene carriers and controls recruited into the TRACK-ON study who had both clinical and neuroimaging data available. This cohort is peri-manifest with 34 of 94 patients having been diagnosed with early Huntington’s disease. In the early-stage Huntington’s disease cohort, the mean TMS was 17.9 (±9.2). See Table 1 for details of the sample.

### Average connectivity parameters show a network-supressing motor cortex activity

**Figure 4:**
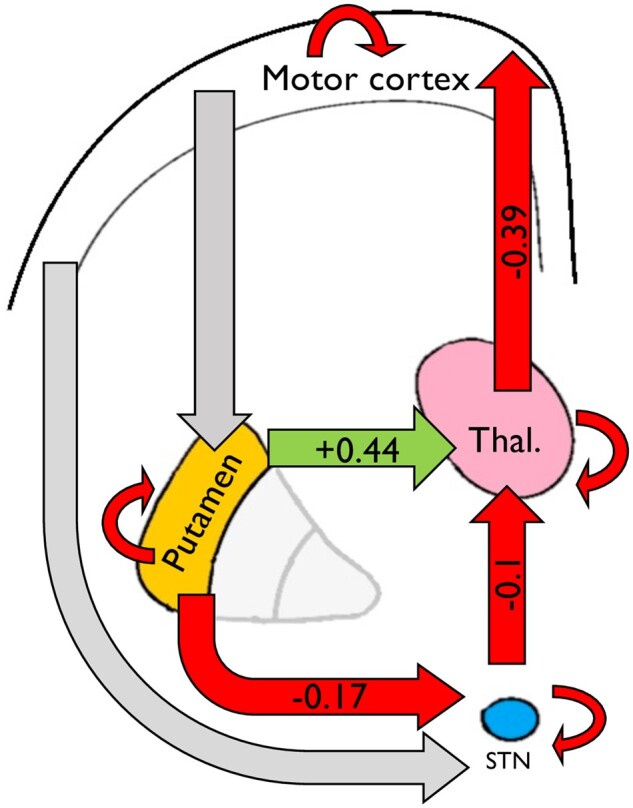
**Schematic showing the average parameter values in the modelled network, across all 94 Huntington’s disease subjects, for between-node connections.** Red arrows indicate suppression of activity, green arrows indicate excitation and grey arrows indicate non-significant connections. Coloured arrows represent connections with a posterior probability of >0.99 for being greater than 0. Overall, the network activity shows a suppression of M1 activity, which may be expected given that subjects are explicitly trying to remain still. Negative self-connections are shown as curved arrows looping back to the node—their values are described in Supplementary Table 1. Model adapted from Kahan *et al*.^36^

During data collection for resting state analysis, participants were explicitly asked to stay still across the scanning session. In keeping with this, average connectivity parameters showed active suppression of the driving input from the motor cortex. The net output from this system via the thalamocortical connection was to supress motor cortical activity (−0.39 Hz, 95% CI: −0.47 to −0.31 Hz, *pp* > 0.99). The ‘direct pathway’ component of our model, the striato-thalamic connection, was found to be excitatory (0.43 Hz, 95% CI 0.37 to 0.50 Hz, *pp* > 0.99). By comparison, the two components of the ‘indirect pathway’ were found to be inhibitory: subthalamic–thalamic (−0.1 Hz, 95% CI: −0.15 to −0.04 Hz, *pp* > 0.99) and striato-subthalamic (−0.17 Hz, 95% CI: −0.24 to −0.11 Hz, *pp* > 0.99). These data are shown in a schematic in Fig. 4 with green arrows representing excitation, red arrows representing inhibition and grey arrows representing non-significant effective connectivity. This connectivity profile was replicated in a cohort of control participants (*n* = 85) from the same study as shown in Supplementary Fig. 3 and Supplementary Table 1. No significant differences between parameter connectivity were found between groups in the between-node connectivity parameters, suggesting that the profile of connectivity replicated in two independent samples.

### Altered connectivity basal ganglia connectivity associated with TMS and apathy scores

**Figure 5:**
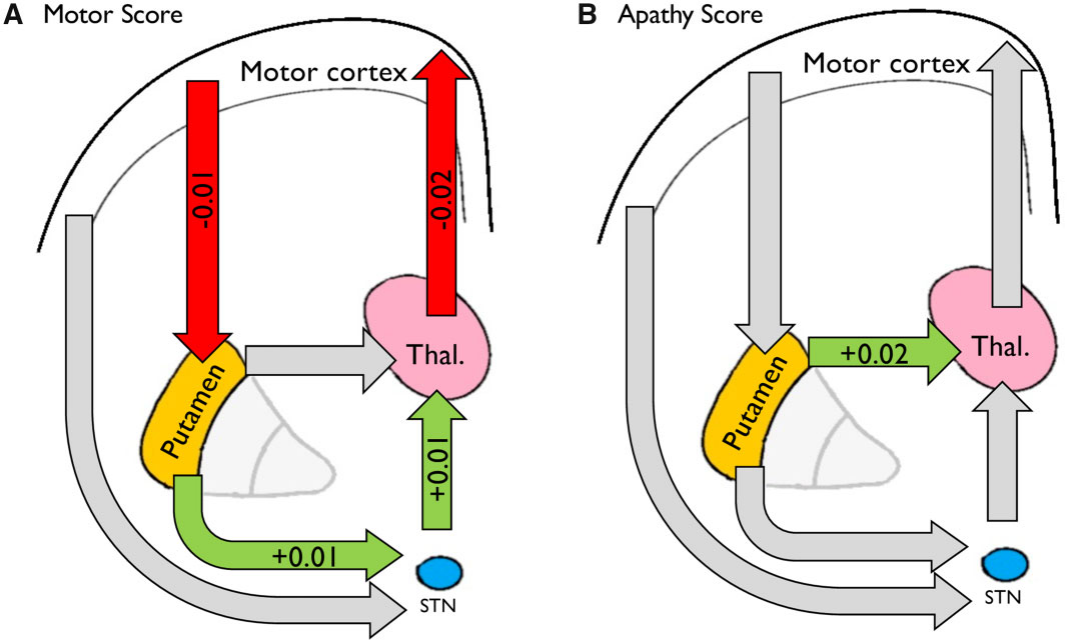
**Association between inter-node connectivity parameters and (A) TMS and (B) Baltimore apathy score.** Green and red arrows indicate which connections were found to be associated with clinical variable with >99% posterior probability using PEB. Grey arrows show connections from the connectivity matrix not found to be associated with clinical scores. Green arrows represent evidence of a positive relationship between connection strength and clinical scores, whereas are arrows represent a negative relationship between clinical score and connection strength.

Using the PEB and BMR procedure (see ‘Materials and methods' section), we tested the hypothesis that changes in connectivity strength within our basal ganglia network would be associated with in TMS and apathy scores. We report only connections found to have very strong evidence (*pp* > 0.99) of being associated with clinical scores. We went on to test whether the strength of these identified connections could predict clinical scores using a leave-one-out cross-validation analysis. The results for motor and apathy analyses are shown graphically in Fig. 5A and B, respectively. Both analyses control for age, sex and scanner type, while apathy analysis also controls for depression and motor score. Results are given as normalized beta values with no units.

#### Motor score associated with changes in indirect pathway connectivity parameters

As shown in Fig. 5A, we found that TMS was positively associated change in both indirect pathway components of our model: striato-STN (0.013, 95% CI: 0.005 to 0.021, *pp* > 0.99) and STN–thalamic (0.011, 95% CI: 0.005 to 0.018, *pp* > 0.99). With reference to Fig. 4, this means that as motor score increased, these connections became less inhibitory.

The weights from the two components of the indirect pathway of our model significantly predicted TMS (*r* = 0.17, *P* = 0.047) in a leave-one-out cross-validation analysis.

TMS was also negatively associated with cortico-striatal connectivity (−0.009, 95% CI: −0.014 to −0.004, *pp* > 0.99) and thalamo-cortical connectivity (−0.019, 95% CI: −0.030 to −0.009, *pp* > 0.99). TMS was positively associated with STN self-connection (0.01, 95% CI: 0.006 to 0.016, *pp* > 0.99).

#### Apathy scores associated change in direct pathway connectivity scores

By comparison, total apathy score was positively associated with strength of the direct pathway component of our model, the striato-thalamic connection (0.022, 95% CI 0.014 to 0.03, *pp* > 0.99). With reference to Fig. 4, this means that as the striato-thalamic connection becomes more excitatory, apathy scores were found to be higher.

Although strong evidence for this effect exists at a group level, weights of the striato-thalamic connection were not strong enough to predict individual apathy scores in a leave-one-out cross-validation analysis (*P* = 0.30)/

Apathy was also negatively associated with STN self-inhibition (−0.013, 95% CI: −0.018 to −0.007, *pp* > 0.99).

The individual parameter estimates by clinical score for both motor and apathy signs are shown in Supplementary Figs 4 and 5, respectively.

## Discussion

We show using functional neuroimaging that motor signs and apathy in Huntington’s disease are associated with unique profiles of altered effective connectivity within basal ganglia pathways. We found strong evidence at a group level that higher motor scores in a large cohort of peri-manifest Huntington’s disease patients were associated with altered coupling in the indirect pathway of our model. By comparison, we identified that apathy scores in prodromal Huntington’s disease may be associated with changes only in striato-thalamic or direct pathway connectivity within our model.

We found that motor signs were associated with less inhibition in the striato-STN and STN–thalamic components of our model, whereas apathy was associated with increased coupling between putamen and thalamus. Given our hypotheses, the motor results are perhaps more intuitive than the apathy results; however, both should be interpreted with caution. Although our hypotheses are based on rate-coding models of striatal function, given the limitation of interpreting blood oxygenation level-dependent signals we do not interpret our results as demonstrating more or less activity in the cell populations we hypothesized. Instead, we simply report evidence that motor signs and apathy were associated with unique basal ganglia connectivity profiles with changes in connectivity associated with each clinical feature mapping onto the connections we hypothesized, within the confines of our model. Our findings may represent a range of pathological processes such as altered rating coding, synaptic dysfunction or altered basal ganglia synchrony.

Although the hypothesis that indirect pathway dysfunction drives the development of motor features of Huntington’s disease is well established, we know of no previous neuroimaging research demonstrating a link between motor score and basal ganglia connectivity in Huntington’s disease research. Furthermore, in this paper we find evidence for novel hypothesis: that apathy in Huntington’s disease may also in part be driven by impaired basal ganglia connectivity, perhaps in the direct pathway. Activity in this pathway drives free-operant movement, a feature commonly lacking in apathy.^14–16^ Computational models of basal ganglia function argue that, via dopaminergic learning signals, the direct pathway cells effectively accrue the value of taking an action.^28–30^ Impaired coupling within this pathway may therefore disrupt both the striatal machinery necessary to take goal-directed actions and the neural representations of the value of those actions. Here we present evidence to support this novel hypothesis.

We believe this study has a number of design strengths. To test our hypotheses, we used data from a large cohort of Huntington’s disease gene carriers who were expressly recruited around the time of motor onset. Many motor signs in Huntington’s disease are not actively elicited and occur at rest—as such, resting state data have considerable ecological validity in trying to understand these features of the disease. The same may be said of apathy. In order to analyse these data, we used spectral DCM, which has been shown to have several benefits when analysing resting state data.^37,^^38^ Using this technique, we found a network whose net output was to reduce activity in the motor cortex. There are few existing data with which to compare these results, but this profile was also replicated in a supplementary control cohort in a separate analysis. We tested the relationship between clinical variables and connections within the network using an advanced Bayesian approach.^39–41^ This procedure compares many competing hypotheses and only those with the strongest evidence survive. As such, our *a priori* predictions were not directly tested but were confirmed *de novo* from the data themselves. In both analyses, we found very strong evidence to support our main hypotheses at a group level. In subsequent analyses to assess translatability to an individual case, we asked whether individual clinical scores could be predicted by the weights of the connections we identified at the group level. Using a leave-one-out cross-validation procedure we found that only motor scores could be predicted from the connections strengths, not apathy scores.

We would also like to draw attention to a few limitations of this study. First, in both analyses we found modest effect sizes. This is perhaps unsurprising. First, in both cases we are sampling from a small region of each structure, and it is unlikely that all clinical change can be attributed to such a restricted region of interest. Second, many neural changes are associated with Huntington’s disease and the pathogenesis of both motor signs and apathy are likely to be biologically heterogeneous. In the case of apathy in particular the literature refers to a range of subtypes—for example, emotional, cognitive, social and behavioural. These subtypes of apathy are believed to have different neurological correlates. Due to the nature of the scale used in this study, we are not able to distinguish these subtypes; however, the scale used weighted it towards behavioural apathy. Furthermore, multiple neurological mechanisms likely contributed to the development of apathy in Huntington’s disease such as white matter changes, involvement of cortical structures or indeed the involvement of other striatal compartments, such as striasomes, which we are unable to currently resolve with *in vivo* imaging.^8,^^57–60^ We therefore do not claim, based on the data presented here, that the changes in connectivity that we present are sufficient to generate clinical features. Rather, we argue that changes in basal ganglia connectivity may contribute to their development in patients.

We should also highlight that we adopted a cross-sectional design. A longitudinal study would give a clearer understanding of the changes that drive the emergence of these features; however, this approach has a number of challenges. Given the slow rate at which clinical features evolve in Huntington’s disease, it is unlikely that longitudinal analysis over a few years would have sufficient power to detect changes in our areas of interest. Instead, we compared across participants with variance in relevant clinical features. We would hypothesize that similar results would be obtained longitudinally if sampled over a longer time period. Our cohort was also in the very earliest stages of manifest disease with low symptom scores. Although this limited the variability in clinical scores, this cohort offered two key advantages. First, very few participants needed to be excluded due to antidopaminergic medication use and second, participants at this stage of disease were able to tolerate functional MRI.

It is also clear that the model used this this study is a simplified model of the relevant basal ganglia circuits. Modelling the true extent of the anatomical complexity within basal ganglia circuits is currently intractable with functional MRI and therefore any attempt to do so requires simplification.^61^ At the core of our model, also used by Kahan *et al*.,^36^ is a connection through which striatal activity can drive thalamic activity directly or via a secondary, indirect route which necessitates striato-diencephalic connectivity in order to change thalamic connectivity.^36^ We found that these pathways excited and inhibited thalamic activity, respectively. On this basis we described them as the direct and indirect pathways in our model; however, we cannot confirm they represent activity in the MSNs as we hypothesize. Although a simplification, we believe this model sufficiently captures the principal dynamics of the network as relevant to the hypotheses we are testing, while also limiting model complexity. Finally, due to the size of the regions we were interested in, especially the STN, partial volume effects are impossible to avoid. However, data extracted from these regions largely conformed to the pattern of activity expected for this system at rest, namely, reduction of motor cortical activity, striato-thalamic excitation via the direct pathway and thalamic inhibition via the indirect pathway. In comparison, previous work treated the STN as a hidden node, meaning that activity from the region is simulated by the model based on *a priori* expected connectivity.^36,^^62^ While avoiding partial volume effects, this approach has the limitation that the model itself must infer the time series from a key node in the network as opposed to modelling data taken from the region itself, the approach taken in this study.

In summary, we demonstrate, using neuroimaging, that changes in connectivity in the basal ganglia motor loop are associated with motor sign severity and apathy in Huntington’s disease. In part, the motivation for this study was to better inform the pathogenesis of these clinical features to advance therapeutics. For apathy in particular, our findings may suggest that medications which manipulate the relative activity of basal ganglia pathways, in particular those that modulate direct pathway activity, may be a fruitful way forward.

## Supplementary Material

awab367_Supplementary_Data

## Abbreviations

BAS: Baltimore apathy scale
BMR: Bayesian Model Reduction
DCM: dynamic causal modelling
FWD: framewise displacement
MSN: medium spiny neuron
PEB: parametric empirical Bayes
STN: subthalamic nucleus
TMS: total motor score
