# Supplementary material for: Imbalanced basal ganglia connectivity is associated with motor deficits and apathy in Huntington’s disease
Source: Brain. 2021 Oct 11;145(3):991–1000. doi: 10.1093/brain/awab367 (PMC9050569; doi:10.1093/brain/awab367)
Supplement: awab367_Supplementary_Data [file awab367_supplementary_data.pdf]

Imbalanced basal ganglia connectivity is associated with motor deficits and apathy in Huntington's disease. Nair *et al* (2021)

Supplementary Material:

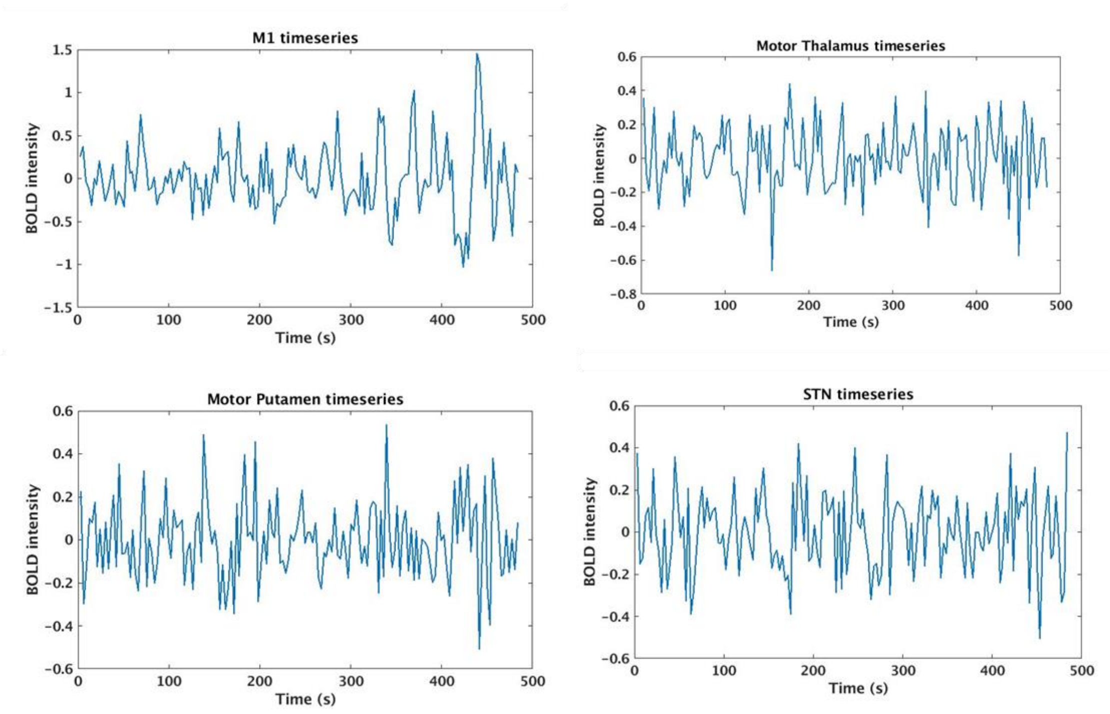

Supplementary Fig (1): Example timeseries from the four ROIs from one participant. Timeseries represent the principle eigenvariate of activity within voxels within each ROI. M1 timerseries is extracted from a 6mm sphere, putamen and thalamus timeseries are extracted from a 4mm sphere and subthalmic nucleus (STN) timeseries is extracted from an age appropriate mask.

Imbalanced basal ganglia connectivity is associated with motor deficits and apathy in Huntington's disease. Nair *et al* (2021)

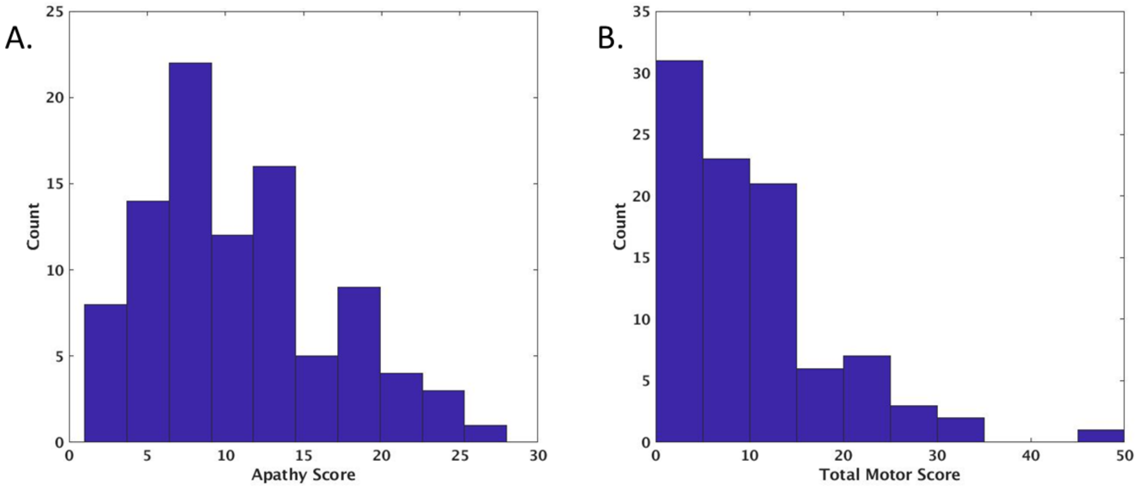

Supplementary Fig (2): A. Spread of apathy scores in the peri-manifest HD cohort as measured by the Baltimore Apathy Scale. Scores on this scale range from 0-42 with higher scores representing higher self-reported apathy. B. Spread of the Total Motor Score in the peri-manifest cohort as measured by the UHDRS total motor score. Scores on this scale range from 0-124. Relatively low scores in this cohort indicate early or premanifest disease.

Imbalanced basal ganglia connectivity is associated with motor deficits and apathy in Huntington's disease. Nair *et al* (2021)

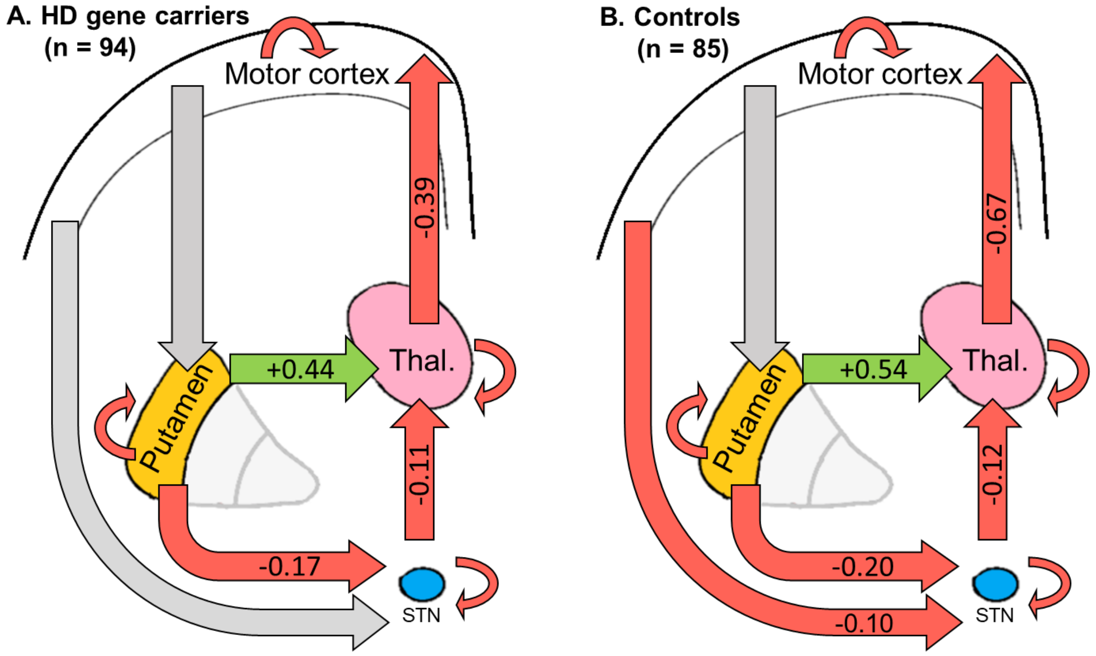

Supplementary Fig (3): Baseline connectivity estimates in the HD gene carriers showed active suppression of motor cortex at rest (model included TMS, age, gender and scanner type as covariates). This connectivity profile was replicated in a cohort of control participants (model included age, gender and scanner type). Parameter estimates with 95% CI and posterior probabilities are show in Supplementary Table 1 for both groups.

Imbalanced basal ganglia connectivity is associated with motor deficits and apathy in Huntington's disease. Nair *et al* (2021)

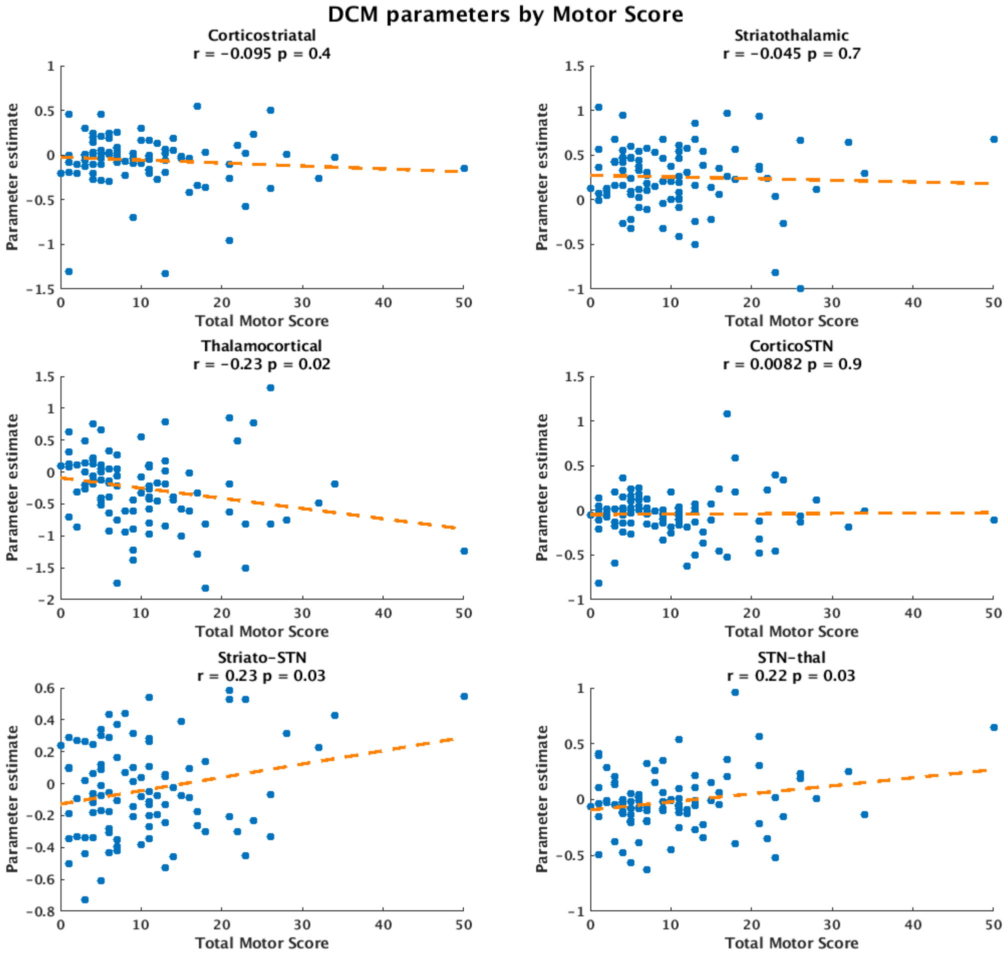

Supplementary Fig. (4): Association between inter-node connectivity parameters and Total Motor Score – individual parameter estimates shown for each HD participant. They show that the connections identified in the hierarchical PEB model mostly also show significant (albeit at uncorrected levels) Pearson correlations between connectivity parameters and clinical scores. It should be noted that not including the covariance here obscures these relationships and the most appropriate demonstration of these results is from the hierarchical PEB model, which being fully Bayesian, not only use the parameters' posterior means but also considers the estimated uncertainty (or variance) resulting in statistically more powerful inference. However, readers may still find these results, using classical inference, of interest.

Imbalanced basal ganglia connectivity is associated with motor deficits and apathy in Huntington's disease. Nair *et al* (2021)

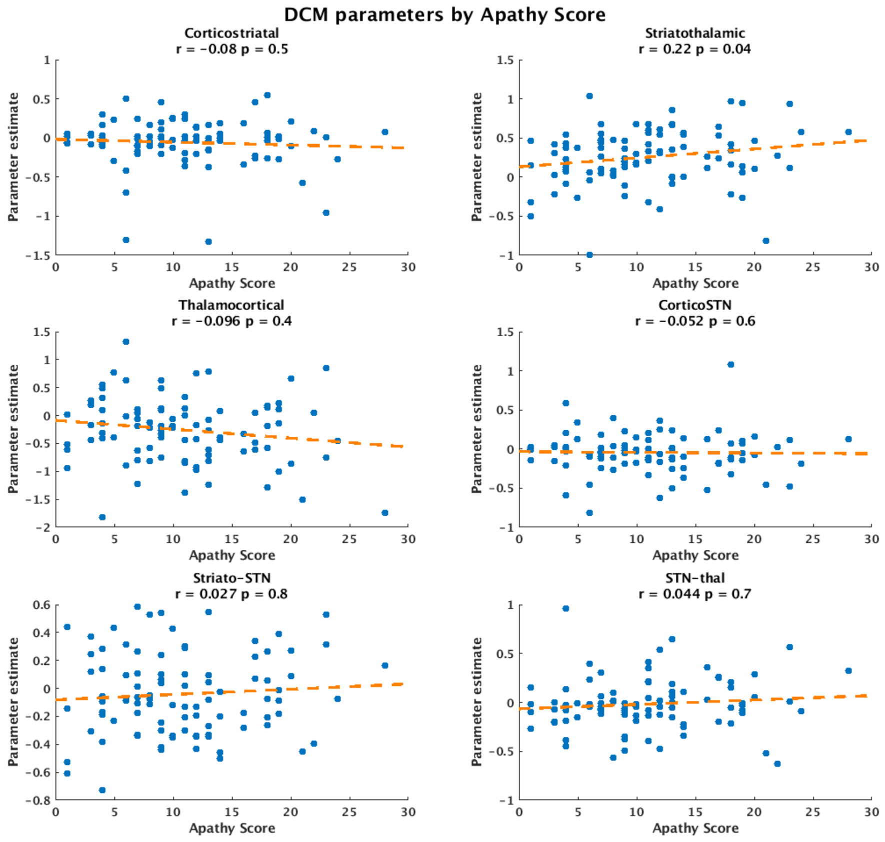

Supplementary Fig (5): Association between inter-node connectivity parameters and Baltimore apathy score (BAS) – individual parameter estimates shown for each HD participant. They show that the connections identified in the hierarchical PEB model mostly also show significant (albeit at uncorrected levels) Pearson correlations between connectivity parameters and clinical scores. It should be noted that not including the covariance here obscures these relationships and the most appropriate demonstration of these results is from the hierarchical PEB model, which being fully Bayesian, not only use the parameters' posterior means but also considers the estimated uncertainty (or variance) resulting in statistically more powerful inference. However, readers may still find these results, using classical inference, of interest.

Imbalanced basal ganglia connectivity is associated with motor deficits and apathy in Huntington's disease. Nair *et al* (2021)

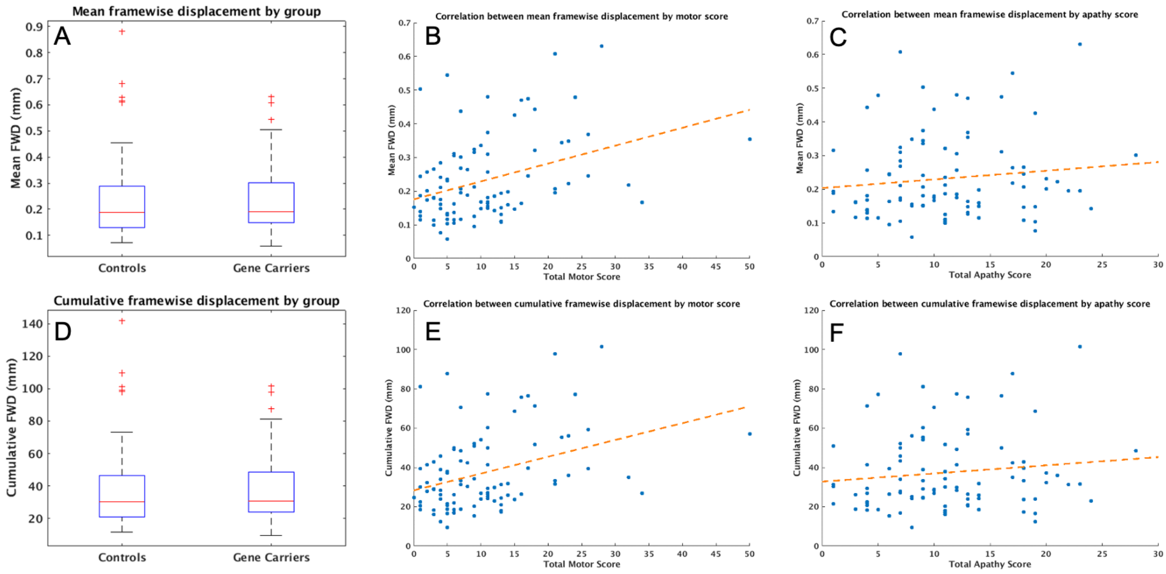

Supplementary Fig (6): A-C show mean framewise displacement as a function of group (A), motor scores (B) and apathy score (C). There was no significant difference between groups ($t(177) = 0.10$, $p = 0.9$) suggesting that additional movement in HD group were not excessive. Unsurprisingly, there was a weak correlation between HD motor score and FWD ($r = 0.36$, $p < 0.01$) and no correlation with apathy ($r = 0.12$, $p = 0.23$). D-F also show the cumulative framewise displacement (total displacement over the entire scan).

Imbalanced basal ganglia connectivity is associated with motor deficits and apathy in Huntington's disease. Nair *et al* (2021)

Average coupling estimates show an active inhibition of M1 in HD gene carriers and controls

	Gene carriers				Controls			
**Connection**	**Mean value (Hz)**	**Lower 95% CI**	**Upper 95% CI**	**Post. prob**	**Mean value (Hz)**	**Lower 95% CI**	**Upper 95% CI**	**Post. prob**
**M1 to motor putamen**	0.00	0.00	0.00	0.00	-0.04	-0.10	0.01	0.73
**M1 to STN mask**	-0.05	-0.12	0.01	0.79	-0.10	-0.15	-0.05	1.00
**Putamen to STN mask**	-0.17	-0.24	-0.11	1.00	-0.20	-0.26	-0.14	1.00
**Putamen to motor thalamus**	0.44	0.37	0.50	1.00	0.54	0.47	0.62	1.00
**STN to motor thalamus**	-0.10	-0.15	-0.05	1.00	-0.12	-0.17	-0.07	1.00
**Thalamus to M1**	-0.39	-0.47	-0.30	1.00	-0.67	-0.77	-0.58	1.00
**M1 self-connection**	0.48	0.40	0.56	1.00	0.64	0.59	0.70	1.00
**Putamen self-connection**	1.16	1.11	1.22	1.00	1.08	1.02	1.15	1.00
**STN self-connection**	1.17	1.13	1.22	1.00	1.12	1.08	1.16	1.00
**Thalamus self-connection**	1.17	1.11	1.23	1.00	1.28	1.23	1.33	1.00

Supplementary Table (1): Comparison of baseline connectivity parameters in the motor network in gene carriers and controls. Both groups show the same overall pattern of effective connectivity (as shown in Supplementary Fig 5) which is suggestive that the motor cortex activity is being suppressed at rest. Positive values of self-connections reflect the degree of self-inhibition. Gene carrier values derive from a model including motor score, age, gender and scanner type. Control values derive from a model including age, gender and scanner type.

The normative control data is presented to demonstrate that using an independent dataset our procedure replicates the same baseline connectivity profile between controls and HD gene carriers. We have formalised this comparison using the PEB contrasts by group – no between node connectivity parameters were found to predict group membership. (note: Putamen (0.177, pp > 0.99) and STN self-connections (0.13 pp > 0.99) were found to be different at a group level however these parameters could not predict group membership using a LOOCV analysis p = 0.59)

Imbalanced basal ganglia connectivity is associated with motor deficits and apathy in Huntington's disease. Nair *et al* (2021)

Supplementary Methods:

Further details of QC from Kloppel *et al* (2015):

The data used in this study was pre-processed as part of the TRACK-ON study and underwent stringent quality control. Although ArtRepair and tsdiffana were used to QC the data, volumes were not removed. Kloppel et al (2015) details the stringent QC as follows:

> "For the raw data, we performed a standard preliminary visual scan of all volumes to check for gross motion artefacts and signal dropouts. Images were then further examined for motion using the ArtRepair Movie function which identifies any artefacts due to sudden motion, global dip, and scanner environment and so on. It is possible to remove volumes using this software, but given the reasons detailed above we did not do this. Instead, we used tsdiffana to investigate variability in signal intensity both between volume and between slice variability; this is similar to the DVARS quality control measure used by Power et al., This enabled us to identify any participants with abnormally high levels of motion marked by abrupt changes in signal intensity either between volume or between slices. Although we did not investigate framewise displacement (between-volume head movement), Power et al have shown that changes in signal intensity are closely associated with between volume head movement prior to pre-processing. Following this, as standard, we ensured that all pre-processing was performed successfully, in particular realignment, co-registration to the structural image and normalisation."
